# Out of School Time Providers Innovate to Support School-Aged Children During the COVID-19 Pandemic

**DOI:** 10.5888/pcd19.210347

**Published:** 2022-03-17

**Authors:** Sarah A. Sliwa, Sarah M. Lee, Laura E. Gover, Danielle D. Morris

**Affiliations:** 1 National Center for Chronic Disease Prevention and Health Promotion, Centers for Disease Control and Prevention, Atlanta, Georgia; 2 Youth Development, Health and Wellness, Boys & Girls Clubs of America, Atlanta, Georgia

The article “Addressing Racial and Ethnic Disparities in COVID-19 Among School-Aged Children: Are We Doing Enough?” described how a shift to virtual schooling strained learning and disproportionately affected school-aged children from racial and ethnic minority groups (1). The authors identified partnerships between school and community organizations, including out of school time (OST) providers (eg, before school, after school, and summer programming), among strategies to curb inequities in health and education that the COVID-19 pandemic exacerbated (1). In this essay, we reflect on how OST organizations exemplified this approach through their responsiveness to challenges that remote learning presented during school year 2020–2021 and how programs continue to support students’ learning and well-being.

## Supporting Students’ Access to Education and Parental Employment With Learning Hubs

Remote learning required that families have reliable access to high-speed internet, a device to connect with learning platforms (eg, smartphone, computer, tablet), and the ability to monitor student engagement. These requirements were not universally met (1). Over 40% of low-income households reported limited access to a computer or broadband internet, and Black and Hispanic households were significantly more likely to report limited access than non-Hispanic White households (2). Some populations that were more likely to be learning remotely were also more likely to lack necessary technology. Monitoring student engagement presented another challenge. Most parents (70%) of a child attending a school with a hybrid schedule were concerned about their ability to juggle work and their child’s remote learning (3). Parents employed as frontline essential workers and single parents may have found it especially difficult to supervise a child learning remotely.

To help families navigate these challenges, some OST providers expanded operations to support remote learning during school hours. OST programs provided students supervised access to technology and tutoring services in addition to offering youths who were in hybrid and fully remote learning with engagement activities during traditional after-school hours. These “learning hubs” took place in after-school programs, such as Boys & Girls Clubs of America (BGCAs), YMCAs, Jewish community centers, parks and recreation sites, and other community settings, including libraries and camps. Some were established in direct coordination with a school district and others through collaboration with an intermediary organization. Some states used relief funds (eg, Coronavirus Aid, Relief, and Economic Security [CARES] Act) to support learning hub operations and provide families with scholarships for more equitable access. The Afterschool Alliance and the National League of Cities developed resources that share applied examples and troubleshooting suggestions, including case studies about partnerships and funding sources. Surveys of OST providers found that 53% served as learning hubs in fall 2020, and 57% in spring 2021 (4,5).

In addition to addressing the digital divide and supporting working parents, these community–school partnerships responded to nonacademic barriers to learning by providing access to healthy foods and beverages, creating opportunities for physical activity, and cultivating a positive social emotional climate (4,5).

## Supporting Access to Nutritious Foods

Shifts in school operations interrupted access to school meals, resulting in fewer breakfasts and lunches served early in the pandemic (March–May 2020) (6). This is concerning because a nutritious and adequate diet supports children’s overall well-being, physical growth, emotional development, and behavior — including the ability to focus while learning (7). The rapid mobilization of free meal sites helped narrow the meal gap (6). Learning hubs served as school meal distribution sites, sponsored meal and snack programs, and partnered with community organizations such as food banks and foundations to provide meals for students’ families during the COVID-19 pandemic. Even as schools return to serving breakfast and lunch on site, OST programs can continue to address food insecurity by providing meals and snacks through federal programs as eligible (eg, Child and Adult Care Feeding Program, National School Lunch Program snacks, Summer Food Service Program), partnering with community organizations, and connecting families with resources.

## Creating Opportunities for Physical Activity

Many sites that served as learning hubs (eg, parks and recreation, YMCAs, BGCAs) had the experience and capacity to organize physical activity opportunities for young people. Programs that provided such opportunities may have helped to buffer documented declines in physical activity among school-aged youths during the COVID-19 pandemic (8). Physical activity offers multiple benefits, including improved fitness, cognition, and reduced symptoms of depression, and may be protective for adolescent mental health during the COVID-19 pandemic (9,10).

## Fostering Connections

OST programs also are known to provide a safe and supervised setting for students (11). During periods of remote learning, learning hubs created a space where youths connected in person with peers (4,5). Children may have benefited from having adults to talk to outside the home because COVID-19 may have placed children at greater risk for abuse or neglect related to factors such as social isolation, increased stress, and job loss (12). As discussed, learning hubs reduced barriers to virtual learning and offered outcomes-based activities, such as tutoring and extracurricular programming (1). To our knowledge, formal evaluations or peer-reviewed articles have yet to quantify the effects of these partnerships on school readiness or student well-being. OST providers and professional organizations that conduct their own data collection activities have an opportunity to communicate their impact to educational partners and decision makers.

## Preventing the Spread of COVID-19

Operating learning hubs and after-school programming incurs some COVID-19 transmission risk because these settings connect(ed) people from different households. Prevention strategies consistent with guidance from the Centers for Disease Control and Prevention (CDC) for K–12 schools, including vaccination for staff and students, can help reduce the risk of transmission. Many OST programs adopted prevention strategies (4,5) and developed resources to support implementation; for example, BGCA’s Back to Club planning includes a physical distancing calculator (see External Resources, at the end of this article).

COVID-19 vaccination among program staff is important for the health of employees, students, and their families and for sustaining program operations. Many after-school programs employ young adults. Since June 2020, COVID-19 incidence has been persistently higher in the 18 to 29 age group and vaccination uptake the lowest among adults (13). Staff with presymptomatic and asymptomatic infections may come to work without knowing they are contagious. Moreover, maintaining adequate staff-to-student ratios and coverage is challenging when staff need to isolate or quarantine. OST programs can support vaccination efforts by providing a frequently asked question (FAQ) document about COVID-19 vaccines for staff and families; collaborating with schools and/or community health partners (eg, local health departments, community centers) to inform families and staff about vaccination events or ongoing clinics; or even hosting a mobile vaccination clinic. Over 180 Boys & Girls Clubs across service areas — military bases, rural, metropolitan/urban, tribal lands, public housing — have served or currently serve as vaccination sites. Many offered clinics alongside traditional back-to-school drives where families could receive critical supplies (backpacks, clothing, computers). Additionally, 117 sites are vaccine education centers. Given high staff turnover in OST settings (14), ongoing efforts to support employee vaccination may be needed.

Taking these precautions requires time, planning, and resources. OST providers will need to continue to assess COVID-19 risk and be prepared to shift modes of operation if needed. Digital, youth-facing platforms (eg, BGCA’s My Future) and guidance for operating virtual programs can be used to sustain connections with caring adults and peers and provide learning support and enrichment activities — especially if programs and schools pivot their operating status to include periods of remote learning (eg, temporary school closures for quarantine).

## Sustaining Partnerships to Support the Whole Child

In CDC’s Whole School, Whole Community, Whole Child Framework (https://www.cdc.gov/healthyschools/wscc/index.htm), which presents a holistic approach to education, the “community” wraps around the model — a reminder that schools do not exist in isolation. The COVID-19 pandemic has further highlighted the interconnectedness of schools and communities. A range of partnerships was initiated and/or strengthened to support students’ physical, emotional, and academic growth and development during periods of distance and hybrid learning — including the development of learning hubs. The BrightSpots database crowdsources innovative collaborations that emerged during the COVID-19 pandemic (see External Resources, at the end of this article).

A widespread return to in-person learning in fall 2021 has decreased the need for learning hubs during school hours. Nevertheless, OST providers and schools continue to serve many of the same students who need reliable access to learning supports, nutritious foods, physical activity, and trusted adults. Recognizing that partnership and coordination remain vital for keeping students safe, supported, engaged, healthy, and challenged, some states are using American Rescue Plan Elementary and Secondary School Emergency Relief (ARP ESSER) to increase funding for summer learning and expanded after-school programing and include OST programs in strategies to recover learning loss (15). These investments have the potential to improve academic, physical, and social–emotional outcomes and narrow disparities among children, adolescents, and young adults. Disruptions to in-person schooling highlighted the importance of OST programs. Recovery efforts now present a unique opportunity to strengthen, sustain, and evaluate the impact of school–OST provider partnerships.

## External Resources

- Afterschool Alliance (2021). Community Learning Hubs: Meeting the Needs of Students & Families. http://afterschoolalliance.org/covid/community-learning-hubs.cfm
- National League of Cities (2020). Municipal Leadership to Support Education Requires Collective Action. Recommendations for building community learning hubs. https://www.nlc.org/wp-content/uploads/2020/10/FINAL20E26EL20Community20Learning20Hub20Brief.pdf
- Boys & Girls Clubs of America. Back to Club. Program basics online planner. https://programbasicsplanner.com/back/
- Forum for Youth Investment (2021). Bright Spots Shining Light on Moving Forward Together: an open-source database capturing the partnerships that support thriving youth. http://brightspots.forumfyi.org/
